# Addressing Adhesive-Induced Agglomeration: Metal Detachment and Flow Behavior in Recycled Paper Fibres/Cellulose

**DOI:** 10.3390/polym17172392

**Published:** 2025-09-02

**Authors:** Sirje Liukko, Katarina Dimic-Misic, Shailesh Singh Chouhan, Michael Gasik

**Affiliations:** 1 Department of Chemical and Metallurgical Engineering, School of Chemical Engineering, Aalto University, 02150 Espoo, Finland; sirje.liukko@aalto.fi (S.L.); michael.gasik@aalto.fi (M.G.); 2 Institute of General and Physical Chemistry Belgrade, 11000 Belgrade, Serbia; 3 CPS-EISLAB, Luleå University of Technology, 97752 Luleå, Sweden; shailesh.chouhan@ltu.se

**Keywords:** recycling paper, deinking of waste paper/recycled paper, mass fraction of heavy metals in recycled paper, plasma mass spectrometry, rheology

## Abstract

This study investigates the presence and potential removal of metal particles that exist in fibers obtained from recycled coated and printed paper, which must be removed through deinking and washing to ensure material safety and optimize pulp formulation for use in food and pharmaceutical packaging applications. For the production of modern packaging material, virgin cellulose fibers are combined with recycled fibers. In such a pulp mixture, recycled fibers introduce sticky particles that contain binders, metals, and ink particles. Those sticky particles that induce aggregation of residues and fibers alter pulp rheology and hinder product formation; therefore, their removal during the deinking process is essential to ensure pulp quality, process efficiency, and product viability. Recycled coated paper was pulped and deinked using a conventional washing process, and the metal content in cellulose pulp was evaluated with Inductively Coupled Plasma Mass Spectrometry (ICP-MS). Rheological measurements were used to reveal its effect on the flow behavior of pulp. The results indicate that the amount of coating with the presence of adhesives and the electronegativity of metals affects metal separation upon washing and deinking. Metals with lower electronegativity, such as Ag, Ti, Cr, V, and Zn, are easily removed from pulp after washing, improving the rheological behaviour of pulp. This research provides novel insights into optimizing the composition and processing of recycled pulp to enhance sustainability, safety, and quality in sustainable packaging production.

## 1. Introduction

In the present effort, the use of biodegradable products for packaging, cellulose occupies first place as the most abundant and recyclable raw material on Earth [1]. Cellulose is a dominant component in the plant cell wall, typically comprising 40–50% in wood, 60% in dried hemp and jute, and reaching its purest form at 90% in cotton. It is a linear polymer constructed from 10,000 D-glucose monomer units. Cellulose obtained from wood and cotton is used in the production of pulp and paper products such as paper, cardboard, cellophane, and textile fibers [1].

Global paper and packaging waste has been steadily increasing due to rising consumer demand and short product life cycles. A significant portion of this waste is recycled to recover valuable fibers and reduce environmental impact [2]. However, recycled paper often contains residual metal particles introduced during coating, laminating, and printing processes [1,2,3]. These metal contaminants can affect the quality and safety of recycled products, especially in applications such as food and pharmaceutical packaging, and therefore need to be removed.

The recycling of paper reduces the environmental impact of paper production, making it a crucial part of the circular economy as global demand for packaging paper rises and the footprint of packages becomes increasingly significant. Within the development of a circular and low-carbon economy, the efficient recycling and reuse of printed office and packaging paper are of paramount importance, and they depend on efficient cleaning of waste products for successful reuse [1,2]. With the increased volume of printed paper waste generated annually [4], the traditional methods in the paper recycling industry are struggling to provide ecological processes and green products [3].

**Figure 1:**
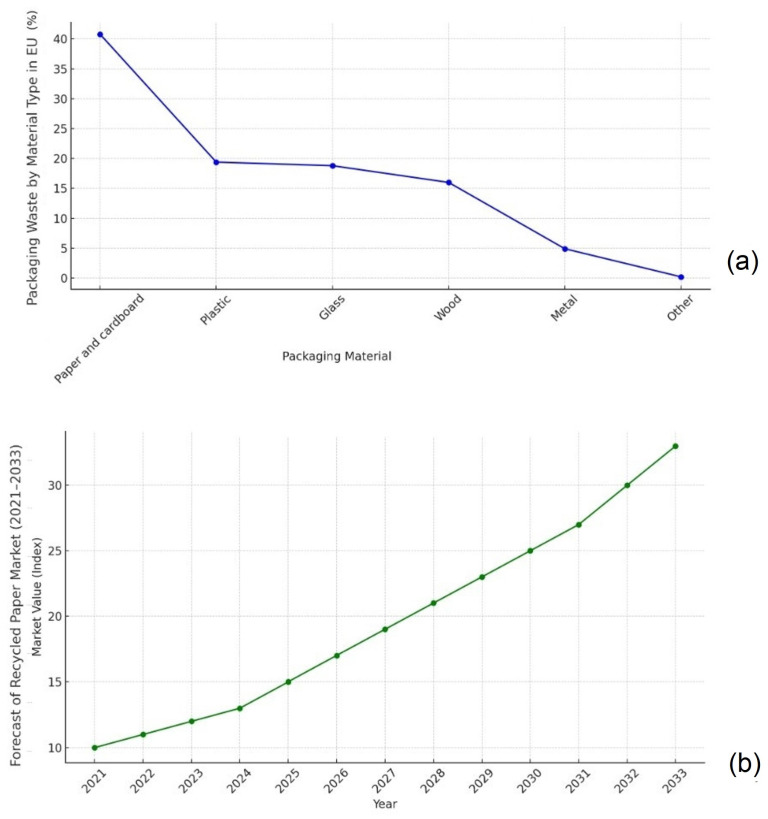
Current trends in development of packaging materials (**a**) paper and cardboard packaging materials are the primary source of packaging (**b**) forecast of the recycled paper and board market 2021–2033. Based on data taken from Eurostat [4].

Cosmetic and food-grade packaging refers to a classification that ensures the safety of the packaging for direct contact with the skin and internal organs [1,5,6]. There are four main types of food and cosmetic-grade packaging: paper, plastic, cans (metal), and glass [7,8]. These materials meet rigorous food safety standards and should be produced according to good manufacturing practices, hazard analysis systems, and regulations [4,9,10]. Eurostat (2024) data indicate that paper and cardboard, already accounting for about 40% of total packaging waste (Figure 1a), are becoming the dominant packaging materials with the rise of the circular economy, and their production from recycled sources is projected to grow exponentially by 2033 (Figure 1b).

One area of particular interest is the recycling of paper packaging containing coating layers and printouts that contain printing inks, which pose unique challenges due to the presence of metallic components [1,5,11,12,13]. In the ever-evolving landscape of security measures, the presence of aluminum in coated paper used for security codes has become a topic of significant interest [6,14,15,16]. The culinary industry has flourished over the recent decade through the emergence of various types of novel culinary businesses and the substantial rise of the food delivery sector in food supply chains, and the development of new circular packaging materials is essential for the sustainability of food and beverage packages [8,17,18]. However, many culinary businesses have not yet fully recognized the importance of fully adopting food-grade safety standards and regulations when it comes to food packaging, while also prioritizing the use of environmentally friendly materials [8,14,19,20].

The increase in consumption and the rise of the global population have increased the use of packaging paper and coated paper that not only improves the visual appearance of products but also provides a barrier against humidity, temperature, dirt, and gases [4,9,20]. Modern packaging often incorporates coated security tags, scanning barcodes, and isolation layers containing metallic elements like aluminum to enhance detection and protection; however, during recycling, these components must be removed to liberate the cellulose polymer from impurities and ensure its suitability for reuse [4,21,22].

As a biodegradable polymer, cellulose fiber-based packaging is a widely used material in the pharmaceutical and food industries [6,22]. Cardboard and paper are nowadays made of reused purified cellulose fibers, and the quality of fibers needs to be such as to ensure excellent printability and coating versatility, enabling brand recognition, marketing visuals, security features, barcodes, and serialization, while also conveying essential product information and usage guidelines [4,23,24].

The incorporation of metals in the inks and polymers used for surface coatings serves to enhance print durability, resistance to tampering, and machine-readable features [12,14,24,25,26]. The properties of polyethylene terephthalate (PET) films used in the packaging industry are significantly influenced by the manufacturing processes employed in their production [16,21,27]. Most PET thin films and sheets are produced by extrusion, where they are stretched sequentially via rolling in the machine direction (MD) and in the longitudinal transverse direction (TD)**,** enabling anisotropic mechanical properties of the product [20,23,28]. This anisotropy, affecting yielding, hardening, and fracture behavior, makes it challenging to accurately model their mechanical behavior and predict failure, which is crucial for their use as a coating substrate for metal-containing coatings [16,27,28,29].

Polymer-containing coating suspensions with embedded metal particles are typically applied as a thin polymer layer roll-bonded to a base sheet [21,28,30]. These composite coatings are widely used across various industries, including packaging, household appliances, automotive components, and construction materials [27,30]. The incorporation of metal particles within the polymer matrix helps prevent corrosion, thereby extending the durability and lifespan of the coatings [14,31,32]. Additionally, the inclusion of pigment particles bound with adhesives enhances the visual appearance of the coated products, often eliminating the need for additional decoration such as stickers or painting [3,12,33,34].

Coated layers also contribute to improved mechanical properties of paper-based substrates, with each additional layer further enhancing the structural strength of the final product [7,35,36,37]. Moreover, advanced coating techniques applied to double-coated substrates significantly enhance the mechanical performance of the coatings, which is an essential factor in the transportation and storage of goods, such as food packaging, cosmetics, and pharmaceuticals [8,17,38,39].

Cellulose, as a biopolymer, is one of the most dominant raw materials in the packaging industry due to its flexibility, availability, biodegradability, and ability to be recycled and reused in numerous production cycles [1,4,8,40,41]. The rapid growth of cellulose demand could potentially lead to increased environmental impacts, including forest cutting, which directly affects biodiversity and contributes to carbon sink depletion, as well as changes in watersheds [40,41,42]. Forests have been increasingly recognized for their role in combating climate change by acting as carbon sinks and supplying sustainable, biodegradable biomass as raw materials [43,44,45].

The use of sustainably managed forest-derived fibers, such as recycled cellulose-based products, has been increasingly recognized by stakeholders as a sustainable alternative to producing virgin fiber materials [45,46,47]. The global success of plastic—rooted in its durability, low cost, and scalability—is now being increasingly challenged by concerns over its environmental and biological impacts, prompting rising interest in alternative materials such as plant-based cellulose, which is emerging as a promising and sustainable competitor [47,48,49].

The published literature indicates that there are several methods for the effective recycling of paper with metal-containing inks, pigments, and coatings [12,26,30,50]. The printed and coated paper and board deinking method utilizes surfactants such as sodium hydroxide (NaOH), sodium sulfate (Na_2_SO_4_), and sodium silicate (Na_2_SiO_3_), which facilitate better contact between inks and water during the washing of printed paper and board, resulting in efficient deinking and the removal of metal particles [3,6,11]. Oleic acid also affects the hydrophilic-lipophilic balance (HLB), which can influence the removal of dye and metal particles from the waste cellulose pulp [3,11,16,38]. The polarity of atoms, which is determined by their electronegativity, may also play a role in separating metal impurities during the deinking process [11,12,51,52]. Enzymatic deinking has been investigated as a sustainable alternative to minimize the environmental impact of chemical-based deinking methods [46,50].

The performance of recycled cellulose from toner-based and inkjet-based prints has been investigated for use in new products, with results showing that pulp recycled from laser-coated paper and board was more effectively purified compared to that from inkjet-coated materials [11,51,52]. This is likely due to the different mechanisms by which the two ink types interact with the paper during the printing and deinking processes [11]. The recycling of paper packaging materials has become increasingly important due to the growing use of paper-based items, including lined paper dishes and cups, driven by the European Union’s ban on certain single-use plastic products [4,53,54,55]. Coated paper and board often contain elevated levels of metals introduced through adhesives, inks, and lamination materials, but deinking has proven effective in reducing metal content in both coated and uncoated substrates, enhancing recyclability and minimizing metallic residues [33,53]. As digital printing and advanced coatings continue to evolve, developing efficient deinking methods that remove printed inks and metal particles is essential to ensure the clean recycling of cellulose fibers, especially for applications requiring high product purity, such as pharmaceutical and food packaging [53,54].

The European Parliament and Council Directive 94/62/EC, enacted on 20 December 1994, mandates that the total concentration of lead, cadmium, mercury, and hexavalent chromium in packaging or its components must not exceed 100 parts per million by weight [55,56,57]. In this research, ICP-MS analysis revealed differences in the amount of metal and metal composition between the non-coated and coated board samples, as well as the effects of the deinking process on metal removal and rheological behavior [57]. The coated samples exhibited higher levels of certain metals, likely due to the adhesives and other materials used in the lamination process [50,57,58,59]. The deinking and washing process was effective in reducing the metal content in both the non-coated and coated samples, indicating the potential for recycling cellulose [57,58,59]. These findings enhance the understanding of the recycling process for coated and printed paper and board, highlighting the impact of metal presence on the circularity and quality of cellulose fiber products [50,53,57,60].

This study aims to investigate the impact of coating and lamination processes, as well as subsequent deinking, with respect to the amount of metal residues remaining in deinked pulp that may be carried over into new recycled paper products. Furthermore, the obtained results and discussion presented in this research provide novel insights into the deinking flotation process and its impact on the extraction of metal impurities from recycled paperboard.

## 2. Materials and Methods

### 2.1. Preparation of Coated Samples

In the present study, we investigate the metal composition of laboratory-made cellulose-based samples derived from non-coated and coated paper prints simulating Chromo cardboard (CG2) printing intended for pharmaceutical packaging [8,25,57]. The base material was laboratory-made hand sheets with a typical weight of 280 g/m^2^ and a thickness of 0.52 mm. The printing substrate was a paper coated with an acrylic polymer, simulating the production of GC2, which is typically coated on the front side and produced in various weights and thicknesses, and used for diverse applications, including hinge-lid boxes, pillow boxes, and counter displays [61].

The paper substrate used in this research complied with standards for food and pharmaceutical products, confirming that it did not contain elevated levels of metals or other hazardous substances, which was the primary focus of this research [54,55,60]. The substrate was designed as a typical product used in modern packaging, comprising a mixture of 50% virgin cellulose pulp fibers and 50% recycled pulp fibers [62,63]. Recycled pulp fibers were obtained from printed coated paper that was deinked and repulped. Deinking was carried out by soaking the paper overnight in water containing 2 *w*/*w*% sodium sulfate (Na_2_SO_4_) relative to the mass of the bone-dry paper, which enhanced the removal of coating and ink particles. The soaked paper was then mixed, subjected to five washing cycles with water, and finally disintegrated by high-shear homogenization [63,64].

The pulps underwent washing cycles and were disintegrated using water in a disintegrator (Lorentzen and Wettre (ABB Group), Kista, Sweden) into a pulp suspension. This suspension was then diluted to 1 *w*/*w*% and mixed in a high-shear Diaf mixer for 30 min. Hand sheets were prepared from the pulp. The upper front side of the substrate received a one- or two-layer coating, whereas the back side was coated just once.

An acrylic polymer-based suspension of polyethylene terephthalate (PET) was used to create the coated printing surface, resulting in a laminate-like layer (BoPET) that meets the standards set by Directive 20/590/EEC [22,33,57,62]. The prints were produced on a coated surface utilizing a five-color offset printer that uses standard UV offset colors, white cover offset printing ink, CMYK inks, and Pantone Matching System (PMS) ink, with a standard printing form on a Roland 705 five-color offset machine press (MAN Roland-R 705, Westmont, IL, USA). The recycled printed and unprinted paper pulp was prepared by mechanical disintegration of the coated substrate, referred to in the text as “coated paper”. The samples were labeled according to the type of paper coating as presented in Table 1.

### 2.2. Inductively Coupled Plasma Mass Spectrometry (ICP-MS)

The paper samples were analyzed for their metal content using Inductively Coupled Plasma Mass Spectrometry (ICP-MS) (PerkinElmer SCIEX™ ELAN^®^ DRC-e, Concord, ON, Canada), a technique well-suited for trace metal analysis. An inductively coupled plasma employs continuous scattering to ionize samples, generating atomic and polyatomic ions for detection [57]. As ICP-MS enables the detection of over 70 elements simultaneously, it was possible to evaluate the presence of Ag, Co, Cr, Cu, Fe, Mn, Ni, Ti, and V.

ICP-MS can detect straightforward spectra with high resolution and very low detection limits, identifying elements at concentrations lower than 0.1 parts per trillion (ppt) and quantifying them in the range of hundreds to thousands of parts per million (ppm) [57,60]. The metals evaluated in this experiment required a detection limit of up to 10 ppt. The ICP-MS system was used to measure the metal content in the coated laboratory paper and recycled cellulose pulp, quantifying element concentrations and providing the total amount for each element of interest. The process involves four stages: sample feed, ICP torch, interface, and MS.

The coated paper samples were cut into small pieces, and 100 mg of each sample was extracted using a hydrochloric and nitric acid solution, as per the protocol of measurement presented in Table 2 and Table 3. Afterwards, the samples were filtered and further diluted before being analyzed by ICP-MS. The system quantitatively measured the concentrations of elements such as Ag, Co, Cr, Cu, Fe, Mn, Ni, Ti, V, and Zn [57,58]. This analytical method enabled a thorough evaluation of the metal composition in the recycled paper samples, considering the effects of the coating process and the deinking and washing treatment on the metal content. Parameters used in ICP-MS are presented in Table 2, while the experimental setup and protocol of measurement are presented in Table 3.

**Figure 2:**
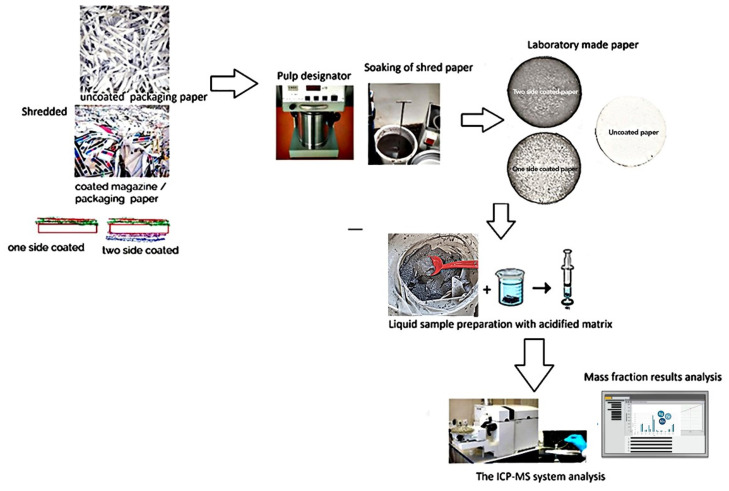
Schematic presentation of experimental design used for ICP-MS metal analysis with recycling and washing of coated sample.

To prepare samples for analysis, both coated and uncoated papers were cut into smaller sections. The samples were soaked in water and defibrillated to disperse the fibers and create a pulp suspension. This pulp was subsequently washed to remove residual particles and ensure consistency for further morphological and surface characterization. A schematic illustration of the experimental setup and sample preparation process is presented in Figure 2.

### 2.3. Agglomerate Size

The agglomerate size was determined using stripped samples disintegrated into pulp, and the measured particle size, i.e., agglomerate size before and after washing, was obtained using dynamic light scattering (DLS), which utilizes the photon correlation spectroscopic technique. Before measuring the particle size defined by the ensemble, the samples were diluted with deionized water to achieve a solid content of 0.01%. The average of five runs was used to obtain the scattering volume equivalent diameter (dsv) and surface charge (ζ) [65].

### 2.4. Rheological Measurements

The rheological investigations were performed at a room temperature of 23 °C using a controlled stress rheometer, Anton Paar 300 (Anton Paar Germany GmbH, Hellmuth-Hirth-Strasse 6, 73760 Ostfildern-Scharnhausen, Germany). Before the measurements, to diminish the effect of disturbances due to sample handling and thixotropy within the suspension, samples were pre-sheared at a shear rate of 100 s^−1^ for 60 s, followed by a resting interval with the same duration. In the viscoelastic region (LVE) prior to critical strain (*γ*c), after which the suspension changes its initial rheological structure, the measurements are considered dependent on the applied strain and the disruption of the overall structure [65,66].

The viscoelastic moduli, storage (elastic) modulus (*G*′)and loss (viscous) modulus (*G*″), were measured using a strain sweep protocol where viscoelastic parameters were detected as a function of strain (γ = 0.01–500%). When LVE was determined, it was established by adopting an amplitude sweep in the oscillatory tests using a constant angular frequency (*ω* = 1 rad s^−1^). In the angular frequency sweep protocol, *G*′, *G*″, and complex viscosity (η*) values were measured as a function of angular frequency (*ω* = 0.1–100 rad s^−1^). All measurements were performed with the logarithmic spread of data points with an increase in strain and a decrease in point duration [64,65].

Measurement under shearing conditions was used for the determination of dynamic viscosity (*η*) flow curves as a function of increasing shear rate (*γ* = 0.01–1000 s^−1^) with a logarithmic spread of data point duration, in the range 100–1 s [66,67,68].

Shear flow measurements were performed using a cylinder geometry with a vane spindle of 10 mm diameter, due to its geometrical arrangement, which enables the breakage of agglomerates and the flocculation of fibers, while also reducing the non-linearity of rheological parameters known as wall slip [66].

Flow curves were plotted in log–log diagrams as dynamic viscosity (*η*) and dynamic stress (*τ*d) in response to shear rate and were fitted to a power law according to the Oswald–de Waele empirical model (Equation (1)), allowing for the comparison of different effects of the amount of pigments and colloidal adhesive residues adhered to pulp fibres:

(1) $$\eta =k{\overset{\cdot }{\gamma }}^{n-1}$$

where *k* and *n* in Equation (1) are the flow consistency index (*k*) and power-law exponent (*n*), and *γ* is the shear rate. Fitting of the flow curve with the Herschel–Bulkley equation (Equation (2)) defines the dynamic yield stress ($\tau_{d}^{0}$) as:

(2) $$\tau =\tau_{d}^{0}+{k\overset{\cdot }{\gamma }}^{n}$$

#### Rheological Data

The variability in rheological measurements for gel-like thixotropic systems remained within 10%, consistent with previously established values for similar systems, with data noise in the raw measurements reduced through exponential smoothing.

## 3. Results and Discussion

### 3.1. Disintegration of Coated Samples

**Figure 3:**
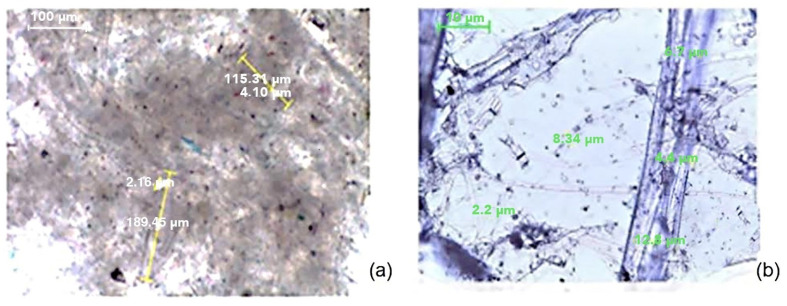
Fiber from deinked pulp; (**a**) one side coated and (**b**) two sides coated paper where visible pigment particles (dark colour) are agglomerated.

Coated paper samples were disintegrated, yielding recycled fibers mixed with agglomerated particles from coated layers and printouts. Some segments consisted of pure recycled fibers, while others retained attached particles of coatings and printing colors. The fibers varied in quality, with some remaining intact and covered in coating particles, while others were broken down into finer fibrils, as shown in Figure 3a,b.

### 3.2. Spectroscopy

**Figure 4:**
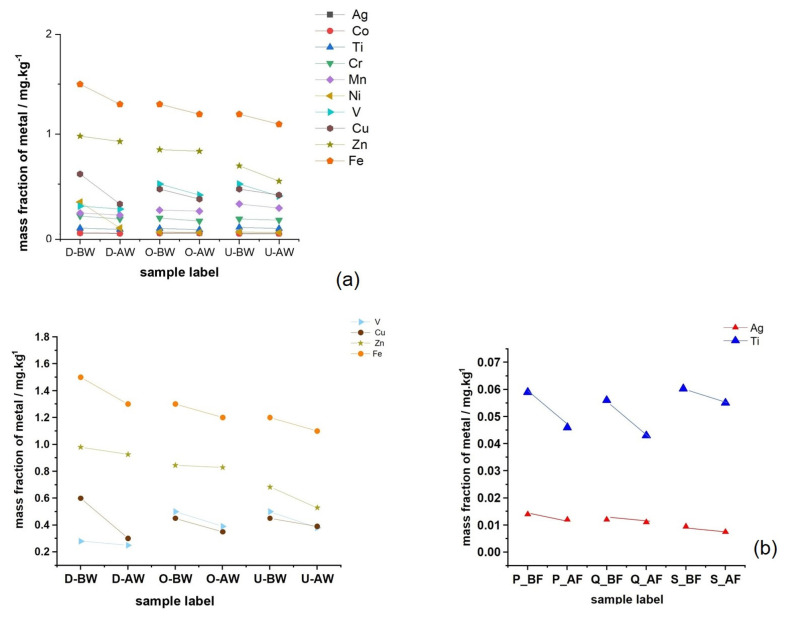
Mass fractions in uncoated and coated samples prior to and after the washing for (**a**) metals with larger fractions and (**b**) the presence of metals in traces.

The mass fractions of metals before and after washing are shown in Figure 4a,b. The results indicate that silver (Ag) concentrations in coated samples were low but slightly higher than those of cobalt (Co), likely due to the contribution of the paper coating. This underscores the influence of coating and printing processes on the distribution of specific metal impurities [11,51,60]. Additionally, the analysis shows that Co was more efficiently removed during the washing process compared to Ag. The results show that Co, with a higher electronegativity than Ag (1.70 vs. 1.42, respectively), exhibited a more significant reduction in mass fraction after washing, suggesting that higher electronegativity may enhance the removal efficiency of metals from cellulose suspensions [57].

Similarly, manganese (Mn), which has a higher electronegativity (1.60) than titanium (Ti), was more effectively separated during the deinking flotation process. Nickel (Ni) was detected in the smallest amounts, with its concentration influenced by the presence of a coating layer, indicating that adhesives can affect metal retention, and that Ni was effectively removed through deinking and washing. For manganese (Mn) and chromium (Cr), there was no significant difference in mass fractions between coated and uncoated samples, although uncoated samples showed slightly higher values. This suggests that deinking and washing effectively remove these metals, irrespective of lamination. Metals Ag and Ti were present with the smallest mass fractions, which decreased after washing (Figure 4b).

**Figure 5:**
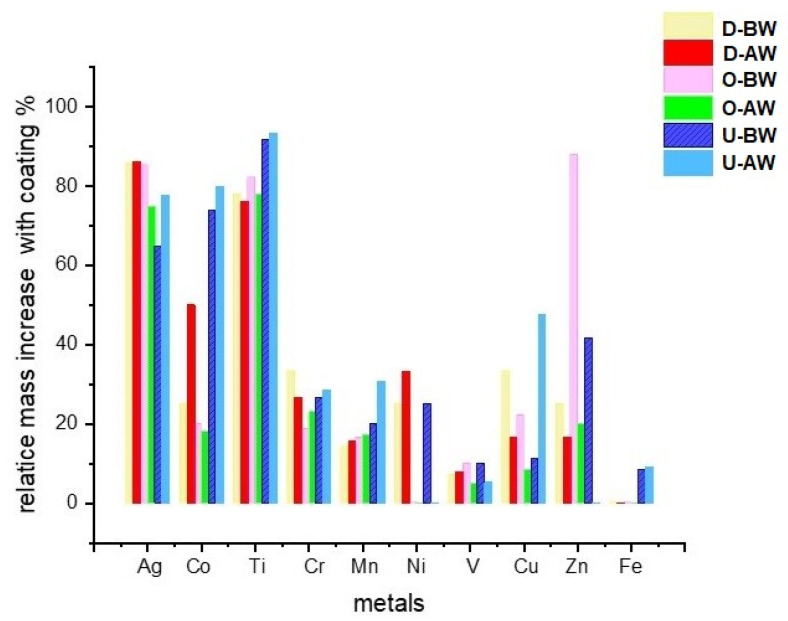
The relative metal content decreased with washing across all three laboratory sheet types—double-sided coated, single-sided coated, and uncoated—both before and after deinking flotation.

The type of adhesive used in paper products likely influences the separation of copper (Cu) and vanadium (V) during the deinking flotation process. As observed previously, the results show that Cu was most effectively removed from non-coated paper, aligning well with the current data in Figure 5 [57,58]. The adhesives used for coating, which are also applied to seal box edges, appear to have minimal impact on the mass fractions of Cu and V before and after pulp washing. However, the lower electronegativity of V, combined with adhesive accumulation at the box edges, may lead to the formation of greater agglomerates, resulting in the agglomeration of V particles after washing. These findings highlight the significant role of adhesive type in the efficiency of metal separation during recycling, which is important for both environmental protection and cost-effective paper recovery.

The metal content in recycled samples decreased after washing, and this reduction was influenced by both the extent of coating and printing, the main sources of metals, as well as the amount of binders [57,58,59]. Binders act as adhesive agents, promoting flocculation and affecting particle agglomeration [67]. Table 4 presents the relative mass fractions of metals that remained on the recycled laboratory papers after deinking and water flushing. After 10 testing cycles, the standard deviation (SD) and variance (σ^2^) for each metal listed in Table 4 were calculated and are summarized in Table 5. These values are reported under different conditions: double-sided coated, one-sided coated, and uncoated paper, both before and after washing of the recycled pulp.

Table 4 shows that the mass fractions of Zn in the samples were slightly higher than those of Fe, and it is evident that Zn concentrations were greater in uncoated samples post-washing—likely due to the lack of adhesive and its lower electronegativity, which may contribute to its reduced extraction and accumulation in the cellulose pulp [68,69]. However, this is not deemed hazardous if the mass fraction stays below 3.5 mg/kg [57]. It should be noted that the presence of adhesive in the drinking water appears to negatively affect the efficiency of Fe removal, which can be due to particle agglomeration within polymer chains and cellulose fiber bundles, as evidenced by the increased iron concentration in coated samples even after deinking through the flotation process [47,63,68]. The mass fractions of samples before and after washing correspond to the traces of printing ink particles.

The analysis of trace metal composition in colloids revealed a wide range of concentrations, from less than 0.1 μg/g to as high as 50 mg/kg of colloidal matter. Interestingly, iron generally had a much higher concentration, typically exceeding 120 mg/kg. While the average concentration for most metals was above 1 μg/g, the concentrations of cadmium, cobalt, and beryllium were below 1 mg/kg.

### 3.3. Particle Size Distribution

Recycled pulp suspensions containing uncoated materials exhibited smaller agglomerate sizes due to the absence of agglomerated ink and adhesive particles. These contaminants typically contributed to foaming during disintegration and interfered with subsequent refining and washing processes [36,47]. In contrast, recycled pulp with a high content of adhesive and coated ink particles tended to form larger agglomerates, which attached to the fibers and further complicated processing [68].

**Figure 6:**
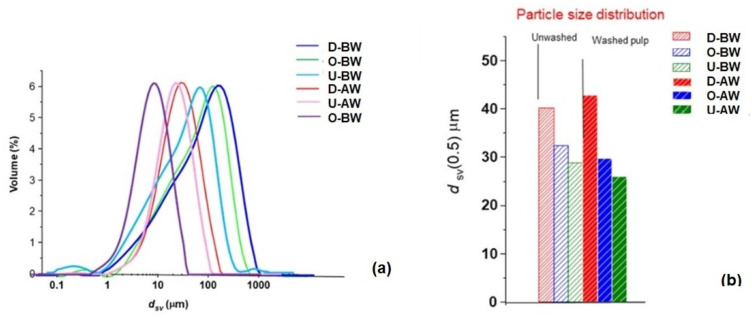
Particle size distribution for uncoated and coated samples before and after washing. Difference in agglomerate size of fibers before and after washing (**a**) particle size distribution and (**b**) average size of agglomerate *d* sv.

From Figure 6a,b, it was revealed that the average agglomerate size of recycled coated samples containing adhesives, ink particles, and fibers decreased with a decrease in applied coating and was reduced further after washing with water. Agglomerate size was the lowest for uncoated paper samples that contained filler particles but no ink particles.

### 3.4. Rheology

Fitting the steady-state curves to the power law model revealed that all recycled pulp suspensions exhibited shear-thinning behavior that progressed with an increase in shear rate, regardless of the presence of ink and adhesive particles in the water [69,70]. Deinked pulp suspensions are non-Newtonian, paste-like fluids that exhibit thixotropic behavior and nonlinear flow curves due to the presence of hydrophobic forces acting between pigment particles and larger agglomerates in the suspension [71].

**Figure 7:**
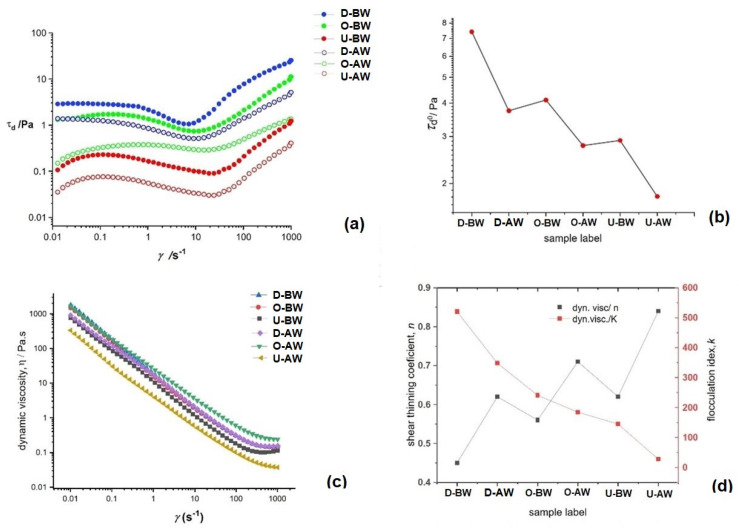
Rheological curves of pulp suspensions (**a**) dynamic stress as a response of shear rate, (**b**) Values of yield values of dynamic stress ($\tau_{d}^{0}$) for all samples, (**c**) dynamic viscosity curves (*η*) as response to increasing shear rate and (**d**) shear thinning index (*n*) and flow index (*k*) of steady state curves for all samples.

The consistency coefficient (k), which denotes the flocculation within the suspension matrix, was higher in suspensions containing larger amounts of metals, inks, and adhesives mixed with cellulose pulp. These additives promote increased agglomeration and flocculation due to more frequent particle collisions and close interactions, leading to dilatant behavior. However, at higher shear rates, liberated pigment particles, mineral fillers, and metals tend to break apart and align with the flow, resulting in shear-thinning behavior across all suspensions [72,73]. The presence of agglomerates adds to the density of the suspension matrix and, through increased flocculation, increases the yield point and the energy necessary to put the suspension into flow motion, as can be seen in Figure 7a,b. Although the flow curves appear similar on a log–log scale, they differ in initial dynamic viscosity (near-zero shear rate at ultralow shear viscosity (η) and slopes of the curves. These differences arise from shear-thickening at higher shear rates due to agglomeration and sticky particles, whereas suspensions with little or no metallic or sticky particles show consistent shear-thinning across the entire range, as presented in Figure 7b,c.

Results of rheological parameters from an average of five measurements are presented in Table 6. Due to the thixotropy of pulp suspensions and flocculation within suspensions, data variation for such systems was within 10%. An increase in agglomeration in suspensions that contained more coated layers and printed ink particles was evident with an increase in the flocculation index k, and it was larger for suspensions that were not washed and for samples that had more coatings on them. Conversely, a smaller amount of coating residues—resulting either from a smaller amount of coatings on the sample or, with washing, a lower amount of sticky polymer agglomerates and ink particles—increases movement between fibers and results in a higher shear-thinning index n. Static yield stress (τs_0_) values were larger for more flocculated suspensions, as more energy was necessary to break flocculates and agglomerates and put suspensions into flow. Therefore, it is evident that uncoated pulp had the lowest yield point. From frequency sweep measurements, we observed that elastic moduli G′ and G″ were largest for more flocculated suspensions and for samples that contained more agglomerates within the fibrous matrix.

## 4. Conclusions

With the growing emphasis on circularity in packaging recycling, the accumulation of metals from coatings, inks, and dyes in recycled pulp poses potential health risks, especially in food and pharmaceutical packaging. This study examines how washing and deinking reduce residual metal content in printed and coated recycled cellulose, assessing its suitability for sensitive applications.

The study found that metal extraction from cellulose pulp obtained from recycled fibers is influenced by both the presence of adhesives and the electronegativity of the metals. We propose that electronegativity is linked to the process characteristics of deinking and washing, which depend on the hydrophilic or hydrophobic nature of the substances involved. Results show that metals such as Ag, Ti, Cr, V, and Zn, which exhibit lower electronegativity, display a smaller increase in mass fraction across certain phases after deinking flotation. Similarly, pulp suspension that has a lower amount of remaining sticky particles and metals in the matrix has better rheological behaviour with pronounced shear thinning behaviour, seen as a higher power law shear thinning coefficient and a lower flocculation index.

These findings highlight the critical importance of understanding the role of adhesives in the formation of sticky particles and the characteristics of metals when designing effective paper recycling processes. Efficient removal of residual adhesives and metals from cellulose is essential not only for improving process performance and material circularity but also for ensuring the safe reuse of recycled fibers in high-value applications such as food, electronics, and pharmaceutical packaging, where purity and safety are crucial.

## Figures and Tables

**Table 1:** Nomenclature of samples used in this research.

Symbol	Sample
D_BW	Double-side coated paper before washing
D_AW	Double-side coated paper after washing
O_BW	One-side coated paper before washing
O_AW	One-side coated paper after washing
U_BW	Uncoated paper before washing
U_AW	Uncoated paper after washing

**Table 2:** Parameters used in ICP-MS Spectrometry.

Parameters Measured	Working Conditions
Spray gas flow rate	0.85 L/min
Auxiliary gas flow rate	1.3 L/min
Plasma flow rate	15 L/min
Lens Voltage	8.5 V
ICP RF Power supply	1100 W
CeO/Ce	0.015
Ba++/Ba+	0.014

**Table 3:** Protocol of experimental setup and detailed description of procedure for ICP-MS Spectrometry measurements.

Stage	Analysis Capability of Elements Analyzing and Characteristics
Sample Feed	Small pieces of coated samples were cut and weighed to about 100 mg.
Chemical Extraction	Five milliliters of hydrochloric acid and nitric acid (1:3 ratio, J.T. Baker, Phillipsburg, NJ, USA, p.a. purity) were added to the sample for metal extraction.
Dilution and Filtration	After extraction, the sample underwent dilution and filtration with a syringe filter, then received an additional tenfold dilution.
Instrumentation	Elemental analysis performed
Analysis Method	An atomized sample generates ions, which are detected.
Detection Capability	Able to identify metals and different non-metals in liquid samples at exceptionally low concentrations.
Elements that were detected	Silver (Ag), Cobalt (Co), Copper (Cu), Chromium (Cr), Nickel (Ni), Iron (Fe), Manganese (Mn), Titanium (Ti), Vanadium (V), Zinc (Zn)

**Table 4:** Relative mass of metals that remained in the laboratory sheets made of recycled paper after deinking and washing.

Metal	Sample Label	Relative Mass of Metal That Was Left on Sample After Deinking and Washing/%
Ti	U_AW	6.73
Ti	U_BW	8.21
Zn	O_BW	12.07
Ag	O_AW	13.79
Ag	D_BW	14.29
Ag	O_BW	14.71
Ti	O_BW	17.86
Co	U_AW	20
Ti	U_BW	22.03
Ti	O_AW	22.09
Ag	U_AW	22.22
Ti	D_AW	23.91
Ag	O_AW	25.12
Co	U_BW	26.32
Ag	U_BW	35.29
Co	D_AW	50.10
Cu	U_AW	52.38
Zn	U_AW	58.33
Ni	D_AW	66.67
Cr	D_AW	66.67

**Table 5:** Results of data variation in mass fraction measurements for metals.

Metal	Condition	SD	σ^2^
Ag	D_BW	0.031	0.025
Ag	D_AW	0.030	0.021
Ag	O-BW	0.020	0.015
Ag	O-AW	0.020	0.015
Ag	U-BW	0.025	0.015
Ag	U-BW	0.020	0.011
Co	D_BW	0.125	0.101
Co	D_AW	0.106	0.101
Co	O-BW	0.085	0.081
Co	O-AW	0.076	0.061
Co	U-BW	0.055	0.031
Co	U-BW	0.036	0.021
Cu	D_BW	0.025	0.001
Cu	D_AW	0.006	0.001
Cu	O-BW	0.015	0.001
Cu	O-AW	0.011	0.001
Cu	U-BW	0.015	0.001
Cu	U-BW	0.006	0.001
Cr	D_BW	0.125	0.081
Cr	D_AW	0.096	0.071
Cr	O-BW	0.055	0.041
Cr	O-AW	0.056	0.041
Cr	U-BW	0.035	0.021
Cr	U-BW	0.026	0.015
Ni	D_BW	0.025	0.001
Ni	D_AW	0.016	0.011
Ni	O-BW	0.025	0.011
Ni	O-AW	0.016	0.011
Ni	U-BW	0.025	0.009
Ni	U-BW	0.016	0.011
Fe	D_BW	0.045	0.032
Fe	D_AW	0.036	0.022
Fe	O-BW	0.025	0.011
Fe	O-AW	0.016	0.012
Fe	U-BW	0.025	0.011
Fe	U-BW	0.016	0.011
Mn	D_BW	0.035	0.021
Mn	D_AW	0.026	0.021
Mn	O-BW	0.025	0.011
Mn	O-AW	0.016	0.011
Mn	U-BW	0.025	0.011
Mn	U-BW	0.016	0.011
Ti	D_BW	0.025	0.001
Ti	D_AW	0.016	0.011
Ti	O-BW	0.025	0.011
Ti	O-AW	0.016	0.004
Ti	U-BW	0.025	0.011
Ti	U-BW	0.016	0.011
V	D_BW	0.055	0.041
V	D_AW	0.032	0.029
V	O-BW	0.025	0.016
V	O-AW	0.032	0.021
V	U-BW	0.025	0.011
V	U-BW	0.022	0.011
Zn	D_BW	0.125	0.078
Zn	D_AW	0.099	0.077
Zn	O-BW	0.085	0.074
Zn	O-AW	0.075	0.068
Zn	U-BW	0.042	0.031
Zn	U-BW	0.032	0.016

**Table 6:** The rheological parameters from an average of five measurements.

	D-BW	D-AW	O-BW	O-AW	U-BV	U-AW
G′_ω = 1.2(rads−1)_ (Pa)	19.45	17.34	14.39	11.45	10.17	8.67
G″_ω = 1.2(rads−1)_ (Pa)	14. 12	12.23	9.88	7.87	8.91	6.63
τ_d_^s^ (Pa)	9.1	7.21	7.56	5.13	5.62	3.23
K (η)	154.65	121.65	139.67	102.45	98.23	76.31
n (η)	0.76	0. 81	0.82	0.85	0.87	0.89

## Data Availability

The original contributions presented in this study are included in the article. Further inquiries can be directed to the corresponding author.

## References

[1] Mandeep, Gupta G.K., Liu H., Shukla P. (2019). Pulp and paper industry–based pollutants, their health hazards and environmental risks. Curr. Opin. Environ. Sci. Health.

[2] Zhou X., Zhou Y. (2015). Designing a multi-echelon reverse logistics operation and network: A case study of office paper in Beijing. Resour. Conserv. Recycl..

[3] Pivnenko K., Eriksson E., Astrup T.F. (2015). Waste paper for recycling: Overview and identification of potentially critical substances. Waste Manag..

[4] EUROSTAT Packaging Waste Statistics, Extracted Data October 2024. https://ec.europa.eu/eurostat/statistics-explained/index.php?title=Packaging_waste_statistics#Waste_generation_by_packaging_material.

[5] Mirkovic I.B., Majnaric I., Bolanca Z. (2015). Ecological Sustainability and Waste Paper Recycling. Procedia Eng..

[6] Purwanto P., Permana-Citra A.D. (2019). Recycling and processing of solid waste into products of the cosmetic packaging industry. J. Phys. Conf. Ser..

[7] Saha T., Hoque M.E., Mahbub T. (2020). Biopolymers for sustainable packaging in food. cosmetics. and pharmaceuticals. Advanced Processing, Properties and Applications of Starch and Other Bio-Based Polymers.

[8] Ibrahim I.D., Hamam Y., Sadiku E.R., Ndambuki J.M., Kupolati W.K., Jamiru T., Snyman J. (2022). Need for sustainable packaging: An overview. Polymers.

[9] Morel S., Mura G., Gallarate M., Sapino S. (2024). Cosmetic Packaging: European Regulatory Aspects and Sustainability. Cosmetics.

[10] Pauwels M., Rogiers V. (2010). Human health safety evaluation of cosmetics in the EU: A legally imposed challenge to science. Toxicol. Appl. Pharmacol..

[11] Karademir A., Aydemir C., Tutak D., Aravamuthan R. (2017). Printability of papers recycled from toner and inkjet-printed papers after deinking and recycling processes. J. Appl. Biomater. Funct. Mater..

[12] Zeltner M., Toedtli L.M., Hild N., Fuhrer R., Rossier M., Gerber L., Raso R.A., Grass R.N., Stark W.J. (2013). Ferromagnetic Inks Facilitate Large Scale Paper Recycling and Reduce Bleach Chemical Consumption. Langmuir.

[13] Bu X., Tabelin C.B., Ulusoy U. (2023). Editorial: Advanced green and sustainable chemical and physical technologies for resources recycling of solid wastes. Front. Chem..

[14] Lamberti M., Escher F. (2007). Aluminium foil as a food packaging material in comparison with other materials. Food Rev. Int..

[15] De Araujo P., Steyer P., Millet J.P., Damond E., Stauder B., Jacquot P. (2003). Pvd aluminium alloy coatings: Environmentally friendly alternative to protect steel parts against corrosion. Surf. Eng..

[16] Bandara R., Indunil G.M. (2022). Food packaging from recycled papers: Chemical, physical, optical properties and heavy metal migration. Heliyon.

[17] Lalpuria M., Anantheswaran R., Floros J. (2020). Packaging technologies and their role in food safety. Microbial Decontamination in the Food Industry.

[18] Sani M.S., Aziz F.A. (2013). Advanced manufacturing systems in food processing and packaging industry. IOP Conf. Ser. Mater. Sci. Eng..

[19] Dos Santos J.M., Quináia S.P., Felsner M.L. (2018). Fast and direct analysis of Cr. Cd and Pb in brown sugar by GF AAS. Food Chem..

[20] Bibi F., Guillaume C., Gontard N., Sorli B. (2017). A review: RFID technology having sensing aptitudes for food industry and their contribution to tracking and monitoring of food products. Trends Food Sci. Technol..

[21] Subedi K., Trejos T., Almirall J. (2015). Forensic Analysis of Printing Inks Using Tandem Laser Induced Breakdown Spectroscopy and Laser Ablation Inductively Coupled Plasma Mass Spectrometry. Spectrochim. Acta Part B Spectrosc..

[22] Olsmats C., Kaivo-Oja J. (2014). European packaging industry foresight study—Identifying global drivers and driven packaging industry implications of the global megatrends. Eur. J. Futures Res..

[23] Bobalek J.F. (2001). Paper products: Security applications. Encyclopedia of Materials: Science and Technology.

[24] Deshwal G.K., Panjagari N.R. (2020). Review on metal packaging: Materials, forms, food applications, safety and recyclability. J. Food Sci. Technol..

[25] Dean D.A., Halli I.H. (2000). An introduction to pharmaceutical packaging. Pharmaceutical Packaging Technology.

[26] Mates J.E., Bayer I.S., Salerno M., Carroll P.J., Jiang Z., Liu L., Megaridis C.M. (2015). Durable and flexible graphene composites based on artists’ paint for conductive paper applications. Carbon.

[27] Soni S.R., Kılıç H., Camden M.P., Derriso M.M., Cunningham S. (2004). Failure analysis and behaviour of titanium alloy metal matrix composite bolted joints. Int. J. Mater. Prod. Technol..

[28] He Q., Wang M., Du Y., Qin Q., Qiu W. (2022). Quantitative characterization of the anisotropy of the stress-optical properties of polyethylene terephthalate films based on the photoelastic method. Polymers.

[29] Feng R., Farris R.J. (2002). Linear thermoelastic characterization of anisotropic poly (ethylene terephthalate) films. J. Appl. Polym. Sci..

[30] Piergiovanni L., Limbo S. (2016). Metal packaging materials. Food Packaging Materials.

[31] Chhipa S.M., Sharma S., Bagha A.K. (2024). Recent development in polymer coating to prevent corrosion in metals: A review. Mater. Today Proc..

[32] Kaliyannan G.V., Velusamy M.K.K., Palaniappan S.K., Anandraj M.K., Rathanasamy R. (2020). Polymer coatings for corrosive protection. Polymer Coatings: Technology and Applications.

[33] Videira-Quintela D., Martin O., Montalvo G. (2021). Recent advances in polymer-metallic composites for food packaging applications. Trends Food Sci. Technol..

[34] Adeyemi J.O., Fawole O.A. (2023). Metal-based nanoparticles in food packaging and coating technologies: A review. Biomolecules.

[35] Son Y.K., Ko D.C., Kim B.M. (2015). Prediction of delamination and tearing during stamping of polymer-coated metal sheet. J. Mater. Process. Technol..

[36] Van den Bosch M.J., Schreurs P.J.G., Geers M.G.D. (2008). Identification and characterization of delamination in polymer coated metal sheet. J. Mech. Phys. Solids.

[37] Carradò A., Faerber J., Niemeyer S., Ziegmann G., Palkowski H. (2011). Metal/polymer/metal hybrid systems: Towards potential formability applications. Compos. Struct..

[38] Kaiser K., Schmid M., Schlummer M. (2017). Recycling of polymer-based multilayer packaging: A review. Recycling.

[39] Huang H.D., Ren P.G., Zhong G.J., Olah A., Li Z.M., Baer E., Zhu L. (2023). Promising strategies and new opportunities for high barrier polymer packaging films. Prog. Polym. Sci..

[40] Sirviö J.A., Liimatainen H., Niinimäki J., Hormi O. (2013). Sustainable packaging materials based on wood cellulose. RSC Adv..

[41] Tajeddin B. (2014). Cellulose-based polymers for packaging applications. Lignocellulosic Polymer Composites: Processing, Characterization and Properties.

[42] Antar M., Lyu D., Nazari M., Shah A., Zhou X., Smith D.L. (2021). Biomass for a sustainable bioeconomy: An overview of world biomass production and utilization. Renew. Sustain. Energy Rev..

[43] Dimic-Misic K., Barcelo E., Brkic V.S., Gane P. (2019). Identifying the challenges of implementing a European bioeconomy based on forest resources: Reality demands circularity. FME Trans..

[44] Perišić M., Barceló E., Dimic-Misic K., Imani M., Spasojević Brkić V. (2022). The role of bioeconomy in the future energy scenario: A state-of-the-art review. Sustainability.

[45] Kam-Biron M., Podesto L. (2011). The growing role of wood in building sustainability. AEI 2011: Building Integration Solutions.

[46] Sahoo K., Bergman R., Alanya-Rosenbaum S., Gu H., Liang S. (2019). Life cycle assessment of forest-based products: A review. Sustainability.

[47] Silviana S., Rahayu P. (2019). Central composite design for optimization of starch-based bioplastic with bamboo microfibrillated cellulose as reinforcement assisted by potassium chloride. J. Phys. Conf. Ser..

[48] Teng C.P., Tan M.Y., Toh J.P.W., Lim Q.F., Wang X., Ponsford D., Lin E.M.J., Thitsartarn W., Tee S.Y. (2023). Advances in cellulose-based composites for energy applications. Materials.

[49] Narancic T., O’Connor K.E. (2019). Plastic waste as a global challenge: Are biodegradable plastics the answer to the plastic waste problem?. Microbiology.

[50] Du X., Lee D.T., Hsieh J.S. (2016). Inkjet ink behaviors and its implication in adsorption deinking. Sep. Sci. Technol..

[51] Mikkilineni A.K., Ali G.N., Chiang P., Chiu G.T., Allebach J.P., Delp E.J. Signature-embedding in coated documents for security and forensic applications. Proceedings of the Security, Steganography, and Watermarking of Multimedia Contents VI, SPIE, Electronic Imaging 2004.

[52] Simske S.J., Aronoff J.S., Arnabat J. Qualification of security printing features. Proceedings of the Optical Security and Counterfeit Deterrence Techniques VI, SPIE, Electronic Imaging 2006.

[53] Bonadies I., Capuano R., Avolio R., Castaldo R., Cocca M., Gentile G., Errico M.E. (2022). Sustainable cellulose-aluminum-plastic composites from beverage cartons scraps and recycled polyethylene. Polymers.

[54] Oloyede O.O., Lignou S. (2021). Sustainable paper-based packaging: A consumer’s perspective. Foods.

[55] European Parliament and Council Directive 94/62/EC of 20 December 1994 on Packaging and Packaging Waste. https://eur-lex.europa.eu/legal-content/EN/TXT/?uri=CELEX%3A01994L0062-20180704.

[56] Coelho S.C., Estevinho B.N., Rocha F. (2021). Encapsulation in food industry with emerging electrohydrodynamic techniques: Electrospinning and electrospraying–A review. Food Chem..

[57] Klemenčić M., Bolanča Mirković I., Bolf N., Markić M. (2024). Determination of the Mass Fractions of the Heavy Metals in the Recycled Cellulose Pulp. Polymers.

[58] Rigol A., Latorre A., Lacorte S., Barceló D. (2002). Determination of toxic compounds in paper-recycling process waters by gas chromatography–mass spectrometry and liquid chromatography–mass spectrometry. J. Chromatogr. A.

[59] Kim-Kang H. (1990). Volatiles in packaging materials. Crit. Rev. Food Sci. Nutr..

[60] Tanase I.G., Popa D.E., Udriştioiu G.E., Bunaciu A.A., Aboul-Enein H.Y. (2015). Estimation of the uncertainty of the measurement results of some trace levels elements in document paper samples using ICP-MS. RSC Adv..

[61] Cirillo G., Curcio M., Spataro T., Picci N., Restuccia D., Iemma F., Spizzirri U.G. (2018). Antioxidant polymers for food packaging. Food Packaging and Preservation.

[62] Gemechu E.D., Butnar I., Gomà-Camps J., Pons A., Castells F. (2013). A comparison of the GHG emissions caused by manufacturing tissue paper from virgin pulp or recycled waste paper. Int. J. Life Cycle Assess..

[63] Gane P., Dimić-Mišić K., Barać N., Imani M., Janaćković D., Uskoković P., Barceló E. (2020). Unveiling a recycling-sourced mineral-biocellulose fibre composite for use in combustion-generated NO x mitigation forming plant nutrient: Meeting sustainability development goals in the circular economy. Appl. Sci..

[64] Ämmälä A., Laitinen O., Sirviö J.A., Liimatainen H. (2019). Key role of mild sulfonation of pine sawdust in the production of lignin containing microfibrillated cellulose by ultrafine wet grinding. Ind. Crops Prod..

[65] Dimic-Misic K., Hummel M., Paltakari J., Sixta H., Maloney T., Gane P. (2015). From colloidal spheres to nanofibrils: Extensional flow properties of mineral pigment and mixtures with micro and nanofibrils under progressive double layer suppression. J. Colloid Interface Sci..

[66] Mohtaschemi M., Dimic-Misic K., Puisto A., Korhonen M., Maloney T., Paltakari J., Alava M.J. (2014). Rheological characterization of fibrillated cellulose suspensions via bucket vane viscometer. Cellulose.

[67] Hubbe M.A., Tayeb P., Joyce M., Tyagi P., Kehoe M., Dimic-Misic K., Pal L. (2017). Rheology of nanocellulose-rich aqueous suspensions: A review. BioResources.

[68] Dimic-Misic K., Nieminen K., Gane P., Maloney T., Sixta H., Paltakari J. (2014). Deriving a process viscosity for complex particulate nanofibrillar cellulose gel-containing suspensions. Appl. Rheol..

[69] Fosmire G.J. (1990). Zinc toxicity. Am. J. Clin. Nutr..

[70] Nikolic M.V., Vasiljevic Z.Z., Auger S., Vidic J. (2021). Metal oxide nanoparticles for safe active and intelligent food packaging. Trends Food Sci. Technol..

[71] Sheng Y., Wang L.T., Sun X.H. (2011). Deinking Technology and Deinking Agent of Waste Paper. Adv. Mater. Res..

[72] Sajjad M., Otsuki A. (2022). Correlation between flotation and rheology of fine particle suspensions. Metals.

[73] Nazari B., Kumar V., Bousfield D.W., Toivakka M. (2016). Rheology of cellulose nanofibers suspensions: Boundary driven flow. J. Rheol..

